# Dietary Strategies for Metabolic Syndrome: A Comprehensive Review

**DOI:** 10.3390/nu12102983

**Published:** 2020-09-29

**Authors:** Sara Castro-Barquero, Ana María Ruiz-León, Maria Sierra-Pérez, Ramon Estruch, Rosa Casas

**Affiliations:** 1 Department of Medicine, Faculty of Medicine and Life Sciences, University of Barcelona, 08036 Barcelona, Spain; sara.castro@ub.edu (S.C.-B.); mariasipe4@gmail.com (M.S.-P.); restruch@clinic.cat (R.E.); 2 Institut d’Investigacions Biomèdiques August Pi I Sunyer (IDIBAPS), 08036 Barcelona, Spain; amruiz@clinic.cat; 3 Centro de Investigación Biomédica en Red, Fisiopatología de la Obesidad y la Nutrición (CIBEROBN), Instituto de Salud Carlos III, 28029 Madrid, Spain

**Keywords:** metabolic syndrome, dietary pattern, Mediterranean diet, plant-based diet, DASH diet, low-carbohydrate diet, high-protein diet, low-fat diet

## Abstract

Metabolic syndrome is a cluster of metabolic risk factors, characterized by abdominal obesity, dyslipidemia, low levels of high-density lipoprotein cholesterol (HDL-c), hypertension, and insulin resistance. Lifestyle modifications, especially dietary habits, are the main therapeutic strategy for the treatment and management of metabolic syndrome, but the most effective dietary pattern for its management has not been established. Specific dietary modifications, such as improving the quality of the foods or changing macronutrient distribution, showed beneficial effects on metabolic syndrome conditions and individual parameters. On comparing low-fat and restricted diets, the scientific evidence supports the use of the Mediterranean Dietary Approaches to Stop Hypertension (DASH) diet intervention as the new paradigm for metabolic syndrome prevention and treatment. The nutritional distribution and quality of these healthy diets allows health professionals to provide easy-to-follow dietary advice without the need for restricted diets. Nonetheless, energy-restricted dietary patterns and improvements in physical activity are crucial to improve the metabolic disturbances observed in metabolic syndrome patients.

## 1. Introduction

Following unhealthy dietary patterns and sedentary lifestyles has led to a notable increase in the prevalence of overweight and obesity worldwide. Non-communicable chronic diseases (NCDs) related to unhealthy dietary patterns and weight gain have expanded in parallel, being the major cause of morbidity and mortality both in developed and underdeveloped countries [1]. Among NCDs, cardiovascular diseases (CVD) and type 2 diabetes mellitus (T2DM) are public health priorities, not only for their high prevalence and outcomes but also for the huge economic burden imposed on the health system [2,3].

Metabolic syndrome (MetS) is a clinical condition characterized by a clustering of metabolic risk factors, which is defined by the simultaneous occurrence of at least three of the following components: central obesity, dyslipidemia, impaired glucose metabolism, elevated blood pressure (BP), and low levels of high-density lipoprotein cholesterol (HDL-c), according to the consensual definition of the International Diabetes Federation, the American Heart Association, and the National Heart, Lung and Blood Institute [4]. In developed countries, the prevalence of MetS has risen up to 20–25% in the adult population, and its incidence continues to increase over time [5,6,7,8]. In Spain, the prevalence of MetS is currently reaching epidemic proportions, affecting approximately 22.7% of the population, taking into account that its incidence increases with age [5]. In addition, MetS increases the risk of T2DM onset and major cardiovascular events by two-fold and five-fold, respectively, and other chronic disease such as cancer, neurodegenerative diseases, non-alcoholic fatty liver disease, the risk of reproductive, lipid and circulatory disorders, atherosclerosis, and all cause-mortality are also increased [8,9,10,11,12,13].

Recent evidence has demonstrated the association between the incidence and prevention of MetS and modifiable lifestyle factors, especially dietary habits. Steckhan et al. analyzed the positive effects of different dietary approaches on MetS inflammatory markers [14]. Regarding the prevention of MetS, Godos et al. also conducted a meta-analysis to demonstrate the preventive role of the promotion of healthy dietary patterns to reduce the prevalence of MetS [15]. Furthermore, some sub-studies from the PREDIMED-Plus cohort showed associations between some dietary components of the traditional Mediterranean Diet (MedDiet) and improvement in MetS components [16,17,18,19,20]. The aim of the present review was to analyze the potential benefits of different dietary approaches on MetS status and their use as efficient strategies to prevent and treat MetS and its comorbidities.

### Dietary Patterns

A single-nutrient dietary intervention has several limitations, and dietary advice must be focused on the overall dietary pattern as part of MetS treatment. Recent evidence supports the implementation of healthy food-based dietary interventions instead of calorie or isolated nutrient restriction [1,21] diets. The health benefits, regarding MetS, of dietary macronutrient patterns and different dietary approaches are summarized in Table 1.

## 2. Mediterranean Diet

The MedDiet refers to the dietary pattern, culture and culinary techniques adhering to countries and populations living in the Mediterranean Sea basin [64]. This dietary pattern has stimulated a great deal of scientific evidence, demonstrating the potential health benefits associated with adherence, and the primary and secondary prevention of many health outcomes, such as CVD, T2DM, and MetS [10,22]. Recent scientific evidence concluded that the MedDiet not only has beneficial effects on health but also has beneficial effects on sustainability and culture [22,65]. Additionally, the MedDiet has been recognized by UNESCO as an Intangible Cultural Heritage of Humanity [66] and the 2015–2020 American Dietary guidelines referred to the MedDiet as an example of a healthy dietary pattern [21]. The MedDiet is a plant-based diet characterized by a high intake of vegetables including leafy green vegetables, fruits, whole-grain cereals, pulses, legumes, nuts, and extra virgin (cold pressed) olive oil (EVOO) as the main source of fat. Moreover, classical recipes are seasoned with sauces such as *sofrito*, whose main ingredients are olive oil, tomato, garlic, onion or leek, rich in phenolic compounds and carotenoids, such as naringenin, hydroxy-tyrosyl, lycopene and β-carotene [67]. Moderate alcohol intake of fermented alcoholic beverages such as red wine, mainly during meals, is also characteristic of the MedDiet, which also comprises a low to moderate intake of fish and poultry, and low consumption of red meat, butter, sweets, pastries and soft drinks [12,23,68]

The traditional MedDiet is a high fat and low-carbohydrate (CH) dietary pattern, which provides a 35–45% of total daily energy intake from fat, about 15% from protein, and 40–45% energy from CH [12,68]. However, the profile of this fat is mainly one of monounsaturated (MU) and polyunsaturated (PU) fatty acids (FA) and the main food sources of total fat intake are EVOO and nuts. EVOO is one of the key foods of the MedDiet and is the main contributor of monounsaturated fatty acids (MUFAs) in MedDiet countries. Oleic acid is the major component of EVOO and many studies have linked MUFA intake to improvements in insulin resistance, one of the main risk factors for MetS, and in blood lipid profile, and a reduction in both systolic and diastolic BP levels [12,24,69]. EVOO is also rich in polyphenols, which present anti-inflammatory and antioxidant effects and contribute to improving the lipid profile and endothelial function [70] Besides the beneficial effects of unsaturated fats, the whole dietary pattern characterized by the high intake of fruits and vegetables together with moderate red wine consumption provides wide nutritional components, such as antioxidant vitamins (vitamin C, E and β-carotene), phytochemicals (such as polyphenols), folates and minerals, which may exert beneficial effects [31,70].

Considering the effects of the MedDiet on MetS, Di Daniele et al. conducted a review addressing the impact of MedDiet adherence on MetS criteria, obesity and adipose tissue dysfunction [10]. The authors reported that prescription of the MedDiet can be used as a possible therapy for MetS, as it prevents the excess of adiposity and obesity-related inflammatory response. Franquesa et al. concluded that there is a strong evidence for the effect of the MedDiet on obesity and on MetS prevention in healthy or high-CVD risk individuals, as well as on the risk of mortality in overweight or obese individuals [22]. As previously cited, a meta-analysis of 12 cross-sectional and prospective cohorts showed that higher adherence to the MedDiet was associated with a 19% lower risk of developing MetS (relative risk (RR): 0.81 (95% confidence interval (CI) 0.71 to 0.92)), and individual components, such as waist circumference and BP, were also improved (RR: 0.82 (95% CI 0.70 to 0.96); RR: 0.87 (95% CI 0.77 to 0.97), respectively) [15]. Several prospective studies observed the same protective effects in Mediterranean and non-Mediterranean countries [25,26,27]. The CARDIA (Coronary Artery Risk Development in Young Adults) study is a prospective study including 4713 individuals which evaluated the evolution of CVD risk factors in black and white populations in the United States [28]. They observed a lower incidence of MetS in individuals with a higher adherence to the MedDiet (Hazard ratio (HR): 0.67 (95% CI 0.49 to 0.90)) compared to those with lower adherence, showing a linear trend according to the five score categories (*p* for trend = 0.005) [25]. Kesse-Guyot et al. conducted a prospective 6-year follow-up with 3232 subjects in the SU.VI.MAX study to evaluate the association between different MedDiet adherence scores and the incidence of MetS. They found that participants with higher adherence had a 53% lower risk compared to the lowest tertile of the MedDiet score (odds ratio (OR): 0.47 (95% CI 0.32 to 0.69) and 0.50 (95% CI 0.32 to 0.77 for each MedDiet score) [26]. In addition, MedDiet adherence scores were associated with improvements in some individual criteria for MetS, such as waist-circumference, BP, triglycerides and HDL-c levels [26]. Moreover, lower MetS prevalence was observed in Korean adults with medium to high MedDiet adherence (OR: 0.73 (95% CI 0.56 to 0.96) and 0.64 (95% CI 0.46 to 0.89), respectively) [30].

MedDiet adherence has been inversely associated with the incidence of CVD and mortality, as well as cancer and degenerative diseases [23,71]. In the case of CVD, the MedDiet is associated with clinically meaningful reductions in the risk of developing the main CVD outcomes, including coronary heart disease and stroke [72]. In a prospective cohort study with 25,994 healthy women from the US Women’s Health Study, Ahmad et al. observed an inverse association between the highest MedDiet adherence score and the incidence of CVD compared to the lowest score (HR: 0.72 (95% CI 0.61 to 0.86), *p* for trend < 0.001) [73]. Among the health effects observed, MedDiet interventions have shown improvements in body composition by reducing total and segmental fat, which might have an effect on metabolic profile [10]. Furthermore, the MedDiet has contributed to a decrease in the incidence of T2DM and CVD, while lessening severity and associated complications in individuals who have already been diagnosed [12,22,23,29,31]. Due to the health benefits associated with this easy-to follow dietary pattern, the MedDiet should be considered as one of the first treatment strategies for the prevention and management of MetS.

## 3. DASH Diet

In 1997, the Dietary Approaches to Stop Hypertension (DASH) diet became a promising strategy for the treatment of high BP [74], and subsequent randomized clinical trials (RCTs) have supported this evidence [32]. This eating pattern promotes vegetables, fruits, whole grains, low- or free-fat dairy products, legumes and nuts intake, while restricting the intake of red and processed meat and sugar-sweetened beverages [74,75]. The DASH diet is characterized by a low-fat content (27% of daily calorie intake from fat), especially saturated fats (6% of energy) and dietary cholesterol (150 mg/d approximately), and reduced sodium content (from 1500 to 2300 mg/day), but it is rich in fiber (>30 g/day), potassium, magnesium and calcium compared to other dietary patterns [55,76]. The DASH diet has proven to be a useful strategy for the treatment of hypertension [32,33,55,77], and several epidemiological studies have associated higher adherence to the DASH diet with a better cardiometabolic profile [34,36,37,38,39,78,79,80]. In a meta-analysis of several cohort studies, Schwingshackl et al. reported that higher adherence to the DASH diet was associated with a significant reduction in the risk of all-cause mortality (RR: 0.78 (95% CI 0.77 to 0.80), the incidence of or mortality by CVD and cancer (RR: 0.78 (95% CI 0.76 to 0.80); RR: 0.84 (95% CI 0.82 to 0.87), respectively) and the incidence of T2DM (RR: 0.82 (95% CI 0.78 to 0.85)) [40].

Regarding the use of DASH diet as an approach for the treatment of hypertension, a recent meta-analysis of 30 RCT with 5545 hypertensive and non-hypertensive participants concluded that the DASH diet together with lifestyle interventions significantly decreased systolic and diastolic BP measurements compared with a control diet (mean differences: −3.2 mm Hg (95% CI −4.2 to −2.3) and −2.5 mm Hg (95% CI −3.5 to −1.5), respectively) [32]. This effect was more pronounced when sodium intake was lower than 2400 mg/d, in subjects under the age of 50, and in participants with hypertension but without antihypertensive medication [32]. Moreover, on comparing the antihypertensive effects of the DASH diet with 13 other eating patterns (including low-fat diet, Nordic diet, MedDiet, Paleolithic diet and low-sodium diet), the DASH diet was the most effective, especially in comparison with low-fat diets [33]. In contrast, Ge et al. identified the Paleolithic and Atkins diets as the most effective dietary patterns for both systolic and diastolic BP management after six months of intervention compared to usual dietary advice, although this effect was not observed after one year of intervention [55].

The DASH diet intervention has also shown potential effects against excess body weight and abdominal obesity [35]. Middle-term dietary interventions have shown a significant reduction in body mass index (BMI) (weighted mean difference: −0.42 kg/m2 (95% CI −0.64 to −0.20)) and waist circumference (−1.05 cm (95% CI −1.61 to −0.49)) [35]. Nevertheless, in overweight or obese individuals, DASH dietary approaches showed significant weight loss compared to other dietary patterns (−3.63 kg (95% Credible Interval −2.52 to −4.76)) whereas this weight loss was lower after one year of intervention (−3.08 kg (95% Credible Interval −0.48 to −5.66)) [55].

The results are not consistent in the case of blood lipoproteins [55,77]. Ge et al. did not observe significant differences in HDL-c or low-density lipoprotein-cholesterol (LDL-c) levels after a DASH dietary intervention versus usual diet [55], whereas in a meta-analysis of 1917 participants with some CVD risk factors, Siervo et al. observed a reduction in total cholesterol and LDL-c levels after the DASH intervention (mean differences: −0.20 mmol/L (95% CI −0.31 to −0.10) and −0.10 mmol/L (95% CI −0.20 to 0.01), respectively), but reported no significant differences in HDL-c and triglyceride levels [77]. Similar results were obtained in a recently published controlled trial in 80 T2DM patients after 12 weeks following the DASH diet compared to an antidiabetic diet based on American Diabetes Association guidelines [81]. Both dietary interventions significantly reduced triglycerides, total cholesterol and very-low-density lipoproteins.

Epidemiological evidence suggests an association between higher adherence to the DASH diet and a better cardiometabolic profile and lower risk of CVD [36,37,38,39]. A cross-sectional study of 1493 adults showed that higher adherence to the DASH diet was associated with 48% less risk of developing MetS, whereas BMI, waist circumference, pro-inflammatory markers and adiposity measures were significantly lower compared to individuals with lower adherence [34]. Interestingly, Ashari et al. observed that higher adherence to the DASH diet was associated with a 64% lower risk of MetS in 425 healthy children and adolescents from 6–18 years of age [82]. In addition, the authors also observed inverse associations among adherence to the DASH diet and BP, fasting plasma glucose levels and abdominal obesity [82]. In this sense, adaptation of the DASH diet to type 1 diabetes glucose requirements (a reduction in CH of around 10% and 15% increase in fat content) resulted in better glucose control and improved the quality of the whole diet, showing a higher intake of fruits, vegetables, fiber and protein compared to the usual intake [83].

The health benefits associated with the DASH diet are probably due to its nutritional quality and distribution. The DASH diet is rich in vegetables and fruits, which translate into high potassium, magnesium and fiber intake, and these nutrients have shown to have a role in BP control, glucose metabolism and insulin response [84]. Furthermore, vegetables and fruits are the main food source of antioxidants and polyphenols, which have been linked to better glucose and insulin blood levels [84]. Moreover, it is limited in sodium and fat, mainly saturated fatty acids (SFA), which are closely related to CVD [84]. Nonetheless, Pickering et al. suggested that the potential health effects of the DASH diet are dependent on eating pattern adherence, with subjects with lower adherence to the DASH diet showing greater benefit from DASH dietary interventions in BP control than those with higher adherence before the dietary intervention [85]. Nonetheless, the commitment and implication of the patient are critical in all life-style interventions based on dietary modifications [86,87].

## 4. Plant-Based Diets

Plant-based diets include a wide variety of dietary patterns, which are characterized by a reduction or restriction in animal-derived food intake and the promotion of plant-source food intake, such as fruits, vegetables, nuts, legumes, and grains. Among plant-based diets, strict vegetarian diets, also known as vegan diets, are defined by the exclusion of all animal-derived products, including dairy products, eggs and honey; lacto-vegetarian diets restrict animal food intake except for dairy products; lacto-ovo-vegetarian diets exclude meat, seafood and poultry but include eggs and dairy products; and pesco-vegetarians or pescatarians are similar to lacto-ovo-vegetarian but include fish [88]. Despite the fact that plant-based diets are defined by the exclusion of some or all animal products, recent evidence defines plant-based diets as dietary patterns that promote a reduction in animal-source food intake along with an increase in plant-based food intake, such as the MedDiet [41,88,89,90].

Plant-based diets have consistently been associated with beneficial cardiometabolic effects, specifically with a lower risk of developing MetS and all of its components [91]. Moreover, these dietary patterns are associated with decreased all-cause mortality and a decreased risk of obesity, T2DM and CVD [43,47,48]. Some studies have found a lower risk of mortality from ischemic heart disease in vegetarians compared with non-vegetarians [43]. Additionally, recent systematic reviews and meta-analyses found significant associations between adherence to the MedDiet and DASH diets and a 38% and 20% lower risk of CVD, respectively, while a 28% reduction in the risk of coronary heart disease was observed following a vegetarian diet [46].

Regarding BP, a meta-analysis of seven RCTs reported a mean reduction of 4.8 mmHg in systolic BP (95% CI −3.1 to −6.6; *p* < 0.001) and a 2.2 mmHg reduction in diastolic BP (95% CI −1.0 to −3.5; *p* < 0.001) in participants following a vegetarian diet compared to an omnivorous diet [42]. These results were confirmed by the same authors in a meta-analysis of 32 observational studies including 604 participants, in which an association was observed between vegetarian diets and reductions in systolic and diastolic BP (−6.9 mmHg (95% CI −9.1 to −4.7; *p* < 0.01) and −4.7 mmHg (95% CI −6.3 to −3.1; *p* < 0.01), respectively) [41,42].

The effects of plant-based diets on blood lipid concentrations are controversial. Wang et al. conducted a meta-analysis of 11 RCTs to evaluate the effects of vegetarian diet on triglycerides, LDL-c, HDL-c and non-HDL-c levels [92]. Total cholesterol levels, LDL-c and HDL-c, were significantly reduced after following a vegetarian diet compared to an omnivorous control diet (0.36 mmol/L (95% CI 0.55 to 0.17; *p* < 0.001), 0.34 mmol/L (95% CI 0.57 to 0.11; *p* < 0.001) and 0.10 mmol/L (95% CI 0.14 to 0.06; *p* < 0.001), respectively). No significant effects were observed for triglyceride levels. This study also described a significant weight-loss in participants who followed the vegetarian compared to the omnivorous diet (−2.88 kg (95% CI −3.56 to −2.20; *p* < 0.001)). Similar results were observed in another meta-analysis of 12 RCTs involving 1151 individuals, in which subjects randomized to the vegetarian diet intervention group showed significant weight loss compared to the non-vegetarian group (mean difference −2.02 kg (95% CI −2.80 to −1.23; *p* < 0.001)) [44]. Other studies assessing plant-based dietary patterns, such as the MedDiet, have also described positive effects on body weight and waist circumference [45].

The health benefits observed are mainly explained by the nutritional quality of plant-based diets as they promote the intake of a wide variety of plant-based foods while cutting down the intake of animal-derived products, such as red and processed meat, which have been associated with a higher risk of developing T2DM, CVD and certain types of cancer [93]. However, it is important to consider that the term “plant-based” does not necessary mean “healthy”, as there is evidence supporting adverse health effects of the excessive intake of some plant-derived foods, such as refined grains, snacks, pastries or sugar-sweetened beverages [41,88,94]. A healthy plant-based diet promotes the intake of whole grains, fruits, vegetables, legumes, and non-hydrogenated vegetable oils, such as EVOO. Thus, plant-based diets have low-energy density and high fiber content, which may contribute to CVD prevention, weight loss and long-term body weight maintenance [41,44,88]. Moreover, the profile of fat is mainly MUFA and polyunsaturated fatty acids (PUFA), while SFA intake is lower compared to other dietary patterns. Replacing SFA by MUFA and PUFA has been linked with anti-inflammatory effects and improvements in insulin sensitivity [88]. Among plant-derived foods, the antioxidant effect exerted by several nutrients and bioactive compounds such as vitamin C and E, β-carotenes and polyphenols has been linked to the prevention of CVD and MetS [41,88,95]. Finally, the replacement of some animal-derived foods implies intake restriction of the harmful components mainly present in red and processed meat, such as excessive sodium, heme iron, nitrates and nitrites, which have been linked to CVD outcomes [41,88,96].

In conclusion, recent evidence has demonstrated the protective effect of plant-based diets against MetS, CVD and their individual risk factors. However, healthy plant-derived food choices are crucial to ensure these beneficial effects. Thus, dietary guidelines should consider healthy plant-based dietary patterns as a potential dietary strategy for the prevention and treatment of MetS.

## 5. Low-Carbohydrate Diet

Low-CH dietary patterns are characterized by a reduction of total CH intake (<50% of daily calorie intake from CH). This type of diet implies a restriction in the intake of several ultra-processed foods, refined grains, starches and foods rich in simple or added sugars [1]. The association between CH intake and the prevalence and management of the MetS is discrepant [97]. In a meta-analysis of 18 studies with 69,554 MetS patients, Lui et al. concluded that the risk of developing MetS was increased in individuals with higher CH intake (2.5% increase in the risk of MetS per 5% energy from CH intake (95% CI 0.4 to 4.8)) [97]. Moreover, some effects on lipid profile were observed in individuals with high CH intake, such as elevated BP, triglycerides and LDL-c and reduced HDL-c levels [98,99]. The mechanisms underlying the health benefits observed in low-CH diets are the avoidance of the rapid absorption associated with some types of CH, such as glucose and refined grains, which leads to an increase in insulin resistance and insulin demand [53,54]. Therefore, in the case of T2DM, recent clinical guidelines do not recommend a specific CH distribution or restriction, and dietary individualization must be prioritized in the treatment and management of this condition [49]. Bazzano et al. conducted a RCT to analyze the effect of a low-CH diet (<40% of total energy intake from CH) compared with a low-fat diet (<30% of total energy from fat, <7% SFA) without energy restriction or physical activity advice in obese adults (BMI 30 to 45 kg/m^2^) [50]. After 1 year of intervention, subjects on the low-CH diet without energy restriction showed greater weight loss (−3.5 kg (95% CI −5.6 to −1.4 kg)), specifically in fat mass (−1.5% (CI −2.6% to −0.4%)). Moreover, some cardiovascular risk factors were improved in the low-CH group, such as triglycerides, HDL-c and total cholesterol to HDL-c ratio [50]. Regarding the management of T2DM, low-CH compared to low-fat dietary interventions (<30% of total energy from fat) showed higher reductions of body weight, glycosylated hemoglobin (HbA1c), triglycerides and BP levels and increased HDL-c concentrations and, consequently, a modification in glucose-lowering medications was observed [49,51].

Recent evidence has shown an association between dietary CH intake and the risk of mortality. The Prospective Urban Rural Epidemiology (PURE) study is a cohort study of 135,335 individuals aged 35–70 years from 18 countries from five continents [98]. The aim of this study was to assess the association of dietary fat and CH intake and total mortality and CVD, differentiating this intake according to the profile of FA and CH. The findings of this study suggest the need for an update in dietary guidelines, with emphasis on fat restriction to promote low-fat and CH dietary patterns (around 50–55% of daily energy intake from CH) rich in PUFA and whole-grain CH. Other studies have also observed an association between refined CH intake and a higher risk of cardiovascular events, such as stroke or myocardial infarction [100,101]. However, there is insufficient scientific evidence on low-CH diets (<50–55% of total energy intake) and metabolic improvements have not been demonstrated in order to support or recommend very-low CH diets [98]. Seidelmann et al. observed that with high (>70% of total energy intake) and low (<40%) CH diets the total mortality increased, with 50–55% showing the lowest risk of mortality, representing a U-shaped association [102]. The replacement of CH with other nutrients has shown different effects on total mortality, which was increased in low-CH diets rich in animal-derived fat and/or protein. By definition, low-CH diets promote the restriction of foods rich in CH, such as vegetables, fruits, whole-grain cereals, legumes, etc. Consequently, low-CH diets, compared to diets with 50–55% of energy from CH, showed lower amounts of bioactive compounds such as fiber, PUFAs, polyphenols, vitamins and minerals [103]. Therefore, the use of very low-CH diets as a dietary approach for MetS should promote plant-based fat and/or protein food sources [102].

Among low-CH diets, it has been postulated that very low CH ketogenic diets have a therapeutic role in several NCDs, including overweight and obesity, CVD and MetS [104]. Although there is no standardized definition of the ketogenic diet, it is characterized by a reduction in CH to less than 10% of daily energy intake, which means around 30 to 50 g of CH per day, and a relative increase of fat intake (fat to CH and protein intake ratio of 3:1 to 4:1) [105]. This restrictive dietary pattern has shown protective effects for obesity and CVD by reducing body weight and improving the lipid profile [104,106,107,108]. The meta-analysis of Bueno et al. observed greater weight loss (weighted mean difference −0.91 kg (95% CI −1.65 to −0.17 kg)), and reduced triglyceride (−0.18 mmol/L (95% CI −0.27 to −0.08)) and diastolic BP levels (−1.43 mmHg (CI −2.49 to −0.37)), while HDL-c levels increased (0.09 mmol/L (95% CI 0.06 to 0.12)) after following a ketogenic diet compared to a low-fat diet [52]. The mechanisms of action underlying these protective effects are as follows: the absence of dietary CH intake leads to a decrease in insulin secretion, which is translated into an inhibition of lipogenesis and fat accumulation and an increase in lipolysis; a satiety effect of protein intake and its effect on appetite control hormones, such as leptin and ghrelin; and the modulation of insulin secretion and ketone body production which might lead to metabolic improvements, especially in insulin signaling [104,109]. Moreover, CH restriction and the glycogen depletion characteristic of this type of diet lead to the use of ketone bodies as the main source of energy. Nevertheless, energy restriction is necessary to maintain ketone body production. Thus, recent evidence suggests that body weight and CVD benefits observed with ketogenic dietary interventions are due to energy restriction, in spite of the macronutrient distribution of the diet [104,110]. However, health care professionals should consider the difficulties in following a ketogenic diet and the absence of healthy foods such as vegetables, fruits and whole-grain cereals, the intake of which is associated with a lower risk of developing chronic diseases such as CVD, T2DM and some types of cancer.

## 6. Low-Fat Diet

By definition, the fat content of the low-fat diet comprises less than 30% of total energy, of which <10% are SFA, with a moderate PUFA content and limited *trans* FA [111,112]. In proportion, CH intake is higher and protein intake is moderate (around 15–17% of total energy intake). Low-fat diets usually include foods and products with reduced total fat content, such as low-fat dairy products instead of whole-fat products and derivatives. Low fat diets in weight-loss oriented dietary interventions showed a reduction in the risk of premature mortality in obese adults [56]. In this sense, a meta-analysis of 34 RCTs observed an 18% lower risk of all-cause mortality in weight-loss oriented dietary interventions in obese adults (95% CI 0.71 to 0.95), while no significant effects were observed in CVD mortality or incidence [56]. Recently, a network meta-analysis described the effectiveness of the low-fat diet for body weight reduction compared to the usual dietary advice and dietary patterns after short-term intervention (6 months), with this effect being attenuated after one year [55].

Clinical trials evaluating the effect of a low-fat diet on the prevalence of MetS have shown conflicting results [113,114,115,116]. Dietary interventions based on low-fat intake (around 20% of total energy intake from fat) slightly reduced MetS components, but no significant effects were observed for CVD or the incidence of coronary heart disease in postmenopausal women compared to the usual diet [113,114]. Nevertheless, following a low-fat diet was not associated with a lower prevalence of MetS in older subjects at high CVD risk [115]. In this sense, Veum et al. compared the effect of a low-fat, high-CH diet (around 30% of total energy intake from fat) vs. a very high-fat, low-CH diet (around 73% total energy intake from fat) on MetS components [117]. No significant differences were observed in MetS components, body weight and body composition in the medium-term [117]. Similar findings were described by Gardner et al. in the DIETFITS trial, in which both low-fat and low-CH dietary approaches showed significant weight loss with no differences between the two interventions [118].

Regarding BP and blood lipoproteins, a low-fat diet showed beneficial effects on systolic and diastolic BP management, and improved HDL-c and LDL-c levels in the short term compared to usual diet, but these effects were reduced in long-term interventions [33,55]. However, in a meta-analysis of RCTs including 17,230 hypertensive and pre-hypertensive participants, Schwingshackl et al. suggested that the MedDiet and the DASH diet are more effective in long-term BP management compared to low-fat diets [33]. Likewise, low-CH diets showed greater effects on the control of glycated hemoglobin and blood lipid levels than low-fat diets in a short to medium term intervention [52,112,119,120]. In the case of glucose metabolism and insulin control, some RCTs have not identified significant effects of low-fat dietary interventions versus other dietary approaches, while higher triglyceride levels were observed, mostly when simple CH proportion is increased [121,122,123,124,125]. In the case of T2DM management, Basterra-Gortari et al. found that a low-fat diet did not have an effect on glucose-lowering medication management while the MedDiet supplemented with EVOO could delay its requirement in older people at high CVD risk [126].

MetS is associated with a pro-inflammatory state, and it has been proposed that a low-fat dietary intervention inducing weight loss slightly reduces inflammatory biomarkers such as high-sensitive c-reactive protein (CRP), interleukin−6 (IL) and tumor necrosis factor alpha (TNF-α) levels [16,127,128,129]. These results are inconclusive, and the effects observed depend on weight loss and diet composition, particularly dietary fiber, fruits and vegetables [16,128]. Additionally, some studies observed that low-fat dietary interventions could improve the microbiome dysbiosis linked to MetS by increasing α-diversity [130,131]. However, limited results are available and more evidence regarding long-term response is needed [132]. Furthermore, nutrigenetic interactions have been described between dietary fat content and metabolic response [133].

Based on the evidence available, current dietary guidelines, such as the 2015–2020 American and European Dietary Guidelines, should avoid stating upper limits of total fat intake, mainly from healthy unsaturated FA. Moreover, this recommendation should include not exceeding 10% of total energy intake from SFA and the replacement of SFA by MUFA and PUFA [22,134].

## 7. High-Protein Diet

Recent evidence suggests that a high-protein dietary pattern leads to greater weight-loss and CVD improvements than standard protein diets (0.8 g protein/kg body weight). High-protein diets are characterized by a 20–30% of daily energy intake from protein, which means around 1.34 to 1.5 g protein/kg body weight [57]. Currently, the use of high-protein dietary interventions has been postulated for the treatment of obesity, MetS and glycemic control [58,93]. The effect of high-protein dietary strategies for weight management is controversial. A meta-analysis of 18 studies on the effect of a high-protein diet in T2DM patients showed that a high-protein diet did not significantly decrease body weight compared to a regular protein diet [135]. Moreover, no significant effects where observed for glycemic control parameters, such as fasting glucose, insulin and HbA1c, blood lipid profile or BP levels. Nevertheless, a significant reduction of triglyceride levels was observed in participants who followed a high-protein diet. In the case of MetS, high-protein diets with CH restriction have shown effective weight-loss in obese adults with MetS [57,58]. Campos-Nonato et al. performed a RCT in 118 adults with MetS to evaluate the effect of a hypocaloric high-protein diet compared to a hypocaloric standard protein diet (500 kcal/day less than the metabolic rate and 1.34 g protein/kg body weight or 0.8 g protein/kg body weight, respectively) [57]. Weight-loss after 6 months of the dietary interventions was significantly higher in participants who followed a high-protein diet (−7.0 kg ± 3.7; *p*-value = 0.046) compared to the standard protein diet (−5.1 kg ± 3.6; *p*-value = 0.157) [57]. MetS criteria, including fasting blood glucose, insulin, homeostatic model assessment for insulin resistance (HOMA-IR) index, and triglyceride, and cholesterol levels, improved in both intervention arms, but non-significant differences were observed in the comparison between groups. The Optimal Macronutrient Intake Trial to Prevent Heart Disease (OmniHeart) study was a randomized, controlled, three-period, crossover nutritional study with 164 participants with overweight or obesity and prehypertension or stage 1 hypertension free of T2DM [136]. This study aimed to evaluate insulin sensitivity with the quantitative insulin sensitivity check index among three dietary interventions: a high-CH diet (58% of daily kcal from CH; 15% from protein and 27% from fat); a protein diet (replacement of 10% of total CH to protein, 25% of daily kcal intake from protein, mainly from plant-based protein sources); and an unsaturated diet (replacement of 10% of total CH to unsaturated fat, 37% of daily kcal intake from fat, mainly from seeds and oils such as olive, canola and safflower oils and nuts). The protein and high-CH dietary patterns did not affect insulin sensitivity, while the unsaturated diet showed improvements in insulin sensitivity, suggesting that the replacement of CH by unsaturated fat, such as in the MedDiet patterns, are alternative dietary approaches to improve insulin sensitivity. The mechanism underlying the potential health benefits of a high-protein diet is that protein induces satiety, which is translated into reduced energy intake in the next meals [137,138]. Furthermore, high protein intake avoids muscle mass loss during energy-restrictive dietary interventions for weight loss [139].

Among protein food sources, meat and meat derived products have been associated with a higher risk of developing T2DM, CVD and MetS [12,13,140]. Dietary guidelines recommend prioritizing plant-based protein food sources such as soy, legumes, beans, nuts and seeds instead of meat and processed meat [21]. Plant-based protein food sources are rich in fibre, phenolic compounds and PUFA, while cholesterol, trans or SFA are in lower proportions [141]. In a recent meta-analysis of 36 RCTs, red meat consumption had no effect on the blood lipid profile or BP, while after analyses stratified by the type of comparison diet, the substitution of red meat with plant-based protein foods showed a reduction in total cholesterol and LDL-c levels [93]. Thus, strong evidence promotes the intake of plant-based protein food sources, and this should also be recommended to promote environmental sustainability.

## 8. Other Dietary Patterns and Strategies

Other dietary alterations have been shown to improve the MetS condition, such as the Nordic Diet, which is characterized by a high content of whole-grain high-fiber products (such as rye, barley, oat, rice, vegetables, fruits and nuts), with rapeseed oil as the main source of dietary fat and a high intake of fish and shellfish [142,143]. Similar to the DASH and the MedDiet, the Nordic diet is considered to be a healthy dietary pattern in that it promotes the intake of vegetables, fruits, fish, poultry, nuts, and is low in sodium, red meat and processed foods. A recent meta-analysis of 5 RCTs including 513 participants demonstrated the effectiveness of the Nordic diet in improving some MetS criteria, mainly systolic and diastolic BP (weighted mean differences −3.97 mmHg (95% CI −6.40 to −1.54; *p* < 0.001); −2.08 mmHg (95% CI −3.43 to −0.72; *p* = 0.003), respectively) [59]. Moreover, improvements in LDL-c (0.30 mmol/l (95% CI −0.54 to −0.06; *p*  =  0.013)), but not in HDL-c and TG levels, were observed compared to control diets [59]. Further studies are needed to evaluate the beneficial effects of the Nordic diet on MetS management and prevention.

Among dietary strategies, intermittent fasting has shown benefits for CVD, T2DM, metabolic disturbances and cancer, mainly because of the daily caloric restriction involved [63]. The main cardiometabolic effects observed after an intermittent-fasting intervention are weight loss and improvements in insulin resistance, dyslipidemia, BP levels and inflammation [60,61,62]. Despite the evidence and potential health benefits of intermittent fasting, the applicability of this dietary strategy is complex and trained health care providers are needed to avoid side effects. Furthermore, De la Iglesia et al. postulated other potential dietary approaches for the prevention and treatment of MetS, such as diets rich in omega−3 FA, low glycemic index, high antioxidant capacity or high meal frequency dietary interventions [144].

Thus, dietary intervention based on energy restriction, independently of the distribution of macronutrients, might influence BP and CVD. Accordingly, most scientific evidence highlights the relevance of dietary quality rather than quantity, especially in the management and prevention of MetS [8,145,146,147]. Moreover, the effectiveness of every dietary intervention is associated with the previous metabolic state (e.g., presence of insulin resistance, T2DM, altered fasting glucose levels, etc.) [148,149]. While multifaceted lifestyle interventions focus on weight-loss and the promotion of physical activity, adherence is the key factor in achieving the beneficial effects observed in each dietary pattern, with intervention adherence being decisive in the results observed independently of the type of diet [150,151,152].

## 9. Conclusions

The protective effects of healthy dietary patterns on MetS seem to be due to the sum of small dietary changes rather than the restriction of any single nutrient. On comparing low-fat diets and very-restricted diets, the scientific evidence supports the use of the MedDiet intervention as the new paradigm for MetS prevention and treatment. The nutritional distribution and quality of the MedDiet allows health professionals to provide easy-to-follow dietary advice without the need for a restricted diet. Nonetheless, RCTs on the effects of a low-CH MedDiet style diet, promoting the intake of whole grain and plant-based protein food sources in patients with MetS, are needed to demonstrate the efficacy of this dietary pattern.

## Figures and Tables

**Table 1:** Dietary strategies and potential health benefits for Metabolic Syndrome (MetS).

Dietary Pattern		Nutritional Distribution	Improvements in MetS Criteria	Ref.
Mediterranean diet		▪ 35–45% kcal/d from total fat (mainly MUFA ^1^, EVOO and nuts being the principal source) ▪ 35–45% kcal/d from CH ▪ 15–18% kcal/d from protein	Reduction of CVD incidence and outcomes	[22,23,24,25,26,27,28,29]
			Decreased BP (systolic and diastolic)	[15,26]
			Inverse association with mortality	[24,30]
			Improvements in dyslipemia	[26]
			Decreased incidence of T2DM	[12,22,23,29,31]
DASH diet		▪ Total fats 27% kcal/d ▪ Saturated fats 6% kcal/d ▪ Dietary cholesterol ▪ CH 55% kcal/d ▪ Proteins 18% kcal/d	Reduction of BP (systolic and diastolic)	[32,33]
			Reduction in BMI and waist circumference	[34,35]
			Improvement in cardiometabolic profile	[36,37,38,39]
			Reduction in T2DM incidence	[40]
Plant-based diets		▪ Reduction or restriction of animal-derived foods ▪ High intake of plant-source foods ▪ Fat profile rich in UFAs	Reduction of BP (systolic and diastolic)	[41,42]
			Decreased body weight and risk of obesity	[43,44,45]
			Reduction of the risk of CVD	[46]
			Decreased all-cause mortality	[43,47,48]
			Decreased risk of T2DM	[43,47,48]
Low CH diets and very low CH diets (ketogenic diets)		▪ <50% kcal/d from carbohydrates and <10% kcal/d from CH in ketogenic diets ▪ High protein (20–30% kcal/d) ▪ High fat intake (30–70% kcal/d)	Weight-loss and weight-loss maintenance	[49,50,51,52]
			Reduction of DBP	[52]
			Reduction of LDL-c and triglycerides levels	[49,50,51]
			Increase of HDL-c levels	[49,50,51]
			Improvements in insulin resistance	[53,54]
			Reduction of HbA1c levels	[49,51]
Low-fat diet		▪ <30% kcal/d from total fat (<10% of saturated fat) ▪ 15–17% kcal/d from protein ▪ 50–60% kcal/d from CH	Reduction of BP (systolic and diastolic)	[33,55]
			Short-term improvement of cholesterol profile	[33,55]
			Short-term weight loss	[55]
			Reduced risk of all-cause mortality	[56]
High protein diet		▪ High protein (20–30% kcal/d) or 1.34–1.50 g/Kg body weight/d from protein ▪ Low CH (40–50% kcal/d)	Reduction of triglycerides levels	[57,58]
Other dietary patterns and strategies	Nordic diet	▪ High content of whole-grain high-fibre products ▪ Low in meat and processed foods	Reduction of BP (systolic and diastolic)	[59]
			Increase of HDL-c levels	[59]
	Intermittent fasting	▪ Fasting for a long period of time	Weight loss	[60,61,62]
			Improvements in insulin resistance	[60,61,62]
			Improvements in dyslipidaemia	[60,61,62]
			Reduction of BP (systolic and diastolic)	[60,61,62]
			Decreased risk of T2DM	[63]
			Decreased risk of CVD	[63]

^1^ Monounsaturated fatty acids, MUFA; extra virgin olive oil, EVOO; carbohydrates, CH; cardiovascular disease, CVD; blood pressure, BP; type 2 diabetes mellitus, T2DM; Dietary Approaches to Stop Hypertension, DASH; unsaturated fatty acids, UFAs; body mass index, BMI; diastolic blood pressure, DBP; low-density lipoprotein cholesterol, LDL-c; high-density lipoprotein cholesterol, HDL-c, glycated hemoglobin, HbA1c; monounsaturated fatty acids, MUFA.
